# From Recognition Lag to the Treatment Gap in Intimate Partner Violence-Related Brain Injury: The Cognitive Entrapment Prospective Framework

**DOI:** 10.3390/brainsci16080863

**Published:** 2026-08-15

**Authors:** James V. English

**Affiliations:** Independent Practice, Helena, MT 59601, USA; drjvenglish@gmail.com

**Keywords:** intimate partner violence, traumatic brain injury, delayed recognition, treatment gap, cognitive entrapment, non-fatal strangulation, rehabilitation, underserved populations, structural barriers, health disparities

## Abstract

**Highlights:**

**What are the main findings?**

- By the time intimate partner violence-related brain injury entered sustained empirical study, the neuroimaging and neuropsychological methods capable of characterizing it were already established. The barrier to recognition was therefore interpretive rather than technological.
- The cognitive entrapment prospective framework recasts apparent staying behavior as a consequence of structural injury to prefrontal-limbic systems involved in planning, prospection, and decision-making, rather than as survivor ambivalence or motivational deficit.

**What are the implications of the main findings?**

- A first immediate step toward narrowing this recognition gap is incorporating brief cognitive screening of working memory and general executive function into clinical assessment of survivors, since observable behavioral compliance may mask structurally driven impairments in the cognitive operations escape requires.
- Sustained progress requires the longer work of revising diagnostic criteria, administrative coding architecture, and population surveillance infrastructure; the cognitive screening step begins recognition immediately while this structural work proceeds.

**Abstract:**

Intimate partner violence-related brain injury is the most recent condition in a 150-year arc in which biological brain injury has been misattributed to psychological or moral causes before formal clinical recognition emerged. Earlier conditions in this pattern were each recognized only after decades of delay. In each prior case, that delay was constrained by limits in available diagnostic technology. Intimate partner violence-related brain injury is the first condition in which diagnostic technology, including computed tomography, magnetic resonance imaging, diffusion tensor imaging, and neurocognitive assessment, has been continuously available throughout the period of non-recognition. This review identifies three structural barriers that sustain this recognition gap: a diagnostic barrier that leaves the injury without formal criteria, an administrative coding barrier that leaves it absent from ICD architecture, and a population surveillance barrier that leaves it indistinguishable from broader assault categories. Each barrier reinforces the others, limiting visibility, resource allocation, and access to care. Across these conditions, this review argues that deferred recognition reflected an institutional dynamic that shaped which injured populations became clinically legible. Recent neuroimaging and cognitive studies make the biological imperative explicit. A cognitive entrapment framework reframes the reduced capacity to engage the cognitive and material resources leaving requires as injury-driven rather than as ambivalence or motivational deficit. The framework offers a mechanistic account of how brain injury may disrupt the multistep planning that leaving demands. Intimate partner violence-related brain injury is not only underdiagnosed but structurally underserved; correcting the mechanisms of recognition failure is necessary for access to treatment and rehabilitation.

## 1. Introduction

### 1.1. Epidemiology and Scale

Intimate partner violence is a pervasive global health problem with durable neuropsychological consequences. The Global Burden of Disease Study 2023 estimated that approximately 608 million women aged 15 and older have been exposed to intimate partner violence in their lifetimes, with intimate partner violence ranking as the fourth leading risk factor for disability-adjusted life years among women aged 15 to 49 worldwide [1]. The World Health Organization’s 2021 global prevalence estimates corroborate this scale, with approximately 27 percent of ever-partnered women aged 15 to 49 reporting lifetime exposure [2]. National prevalence estimates reach 34 to 40 percent in the United States and Canada [3,4].

Studies indicate that 40 to 92 percent of survivors report blows to the head or face, and 30 to 68 percent report strangulation, a mechanism strongly associated with hypoxic–ischemic injury [5,6,7]. These neurobiological exposures occur concurrently with acute threat responses and chronic traumatic stress, creating a complex clinical presentation in which cognitive inefficiency, slowed processing, and attentional dysregulation are readily misattributed to psychological trauma alone.

### 1.2. The Emerging Literature

Work has begun establishing intimate partner violence-related brain injury as a distinct clinical entity. Kwako and colleagues’ early review charted the field’s initial scope [8]. Esopenko and colleagues synthesized evidence across five priority areas: study design, mechanisms of non-fatal strangulation, biomarkers, chronicity, and brain injury as a risk factor for intimate partner violence engagement [9]. Subsequent investigations have documented white matter differences associated with repetitive intimate partner violence-related traumatic brain injury [10], memory and learning differences persisting beyond initial injury [11,12], and vestibular dysfunction associated with cumulative injuries [13].

The literature has given comparatively little attention to why these injuries remained outside formal clinical recognition for roughly four decades after the diagnostic technology became available. The empirical foundation dates to Valera and Berenbaum’s 2003 demonstration that battered women sustain measurable brain injuries with corresponding cognitive deficits; an earlier shelter-based inquiry had already documented a high prevalence of head injury among battered women [14], and contemporaneous emergency-department screening work documented the same under-recognized association [15]. Twenty years later, intimate partner violence-related brain injury was absent from the 2023 American Congress of Rehabilitation Medicine diagnostic criteria, the field’s nosological standard.

This prolonged non-recognition reflects a broader historical pattern in which cognitive impairment has been misattributed to dispositional or motivational factors rather than neurobiological constraints. Suchy’s contextual framework of executive functioning bears directly on this misattribution [16,17]. The framework demonstrates that cognitive performance reflects dynamic interactions between neurobiological limitations and situational demands, not stable personality traits. This framework helps explain how injury-driven cognitive constraints in survivors were reframed as ambivalence, noncompliance, or psychological reluctance, thereby obscuring the underlying neuropathology and delaying formal clinical recognition.

### 1.3. The Recognition Lag and Structural Barriers

A consistent pattern runs through medicine’s engagement with neurologically invisible injury. The interval between a population’s first documented exposure to a mechanism of brain injury and that population’s formal clinical recognition has been measured in decades, not years [18,19,20,21]. The pattern held for railway accident survivors in the 1860s, for combat veterans across two world wars, and later for boxers and professional football players. What drove the delay was not the absence of technology. Computed tomography was in clinical use by 1971 and structural magnetic resonance imaging by the early 1980s, yet recognition was deferred in each succeeding era even as these tools became available. The mechanism is institutional. Recognition has depended on social conditions: whose injuries commanded attention, and which professions held the authority to formalize them in clinical language.

Intimate partner violence-related brain injury follows the same pattern with one distinction. The full diagnostic and neuroimaging suite has been available throughout the period of non-recognition and has been actively deployed on this population, generating measurable findings, for more than two decades [10,11]. Here, the limiting factor has not been technological. Recognition has remained obstructed by the same institutional and interpretive constraints that operated in earlier periods, now without the technological justification that applied before.

Three interdependent barriers account for the persistence of this recognition gap in the contemporary period. None reflects system failure in any conventional sense; each reflects a system built around a taxonomy that did not anticipate dual-mechanism injury in the IPV context. The first is historical and clinical. Psychological frameworks that emerged when biological understanding of intimate partner violence-related brain injury was unavailable, including learned helplessness, traumatic bonding, and codependency, occupied the explanatory space and remain operative in shelter and clinical settings.

The second is nosological. The 2023 revision of the American Congress of Rehabilitation Medicine diagnostic criteria for mild traumatic brain injury, intended to expand and update the field’s standard, did not include intimate partner violence-related brain injury as a qualifying mechanism. Strangulation-induced hypoxic–ischemic injury, the dominant second mechanism of intimate partner violence-related brain injury, was not enumerated among qualifying mechanisms [22,23].

The third is structural. The surveillance and administrative coding infrastructure that would generate the population-level data capable of forcing nosological revision is itself organized around external causes that do not separate intimate partner violence from other assault categories [9,24,25,26]. A population that cannot be counted cannot generate the epidemiological pressure that forces the systems above it to change.

The cognitive entrapment framework offered here draws on two converging empirical lines to specify a mechanistic account of goal-directed behavior under repetitive brain injury. Suchy’s contextual model of executive functioning establishes that performance is not a trait but the dynamic product of neurobiological substrate, current state, and situational demands [16,17]. Valera and colleagues have established a parallel empirical line in intimate partner violence-related brain injury, documenting measurable degradation in memory, learning, and cognitive flexibility scaled to injury severity [9,11], with neuroimaging evidence of disrupted large-scale network dynamics between salience and default-mode systems central to attentional control and goal-directed action [27]. Read together, these lines specify what the framework targets: the frontal-system substrate on which adaptive behavior depends, including the planning, sequencing, prospection, and flexible revision of action that escape-related decision-making demands.

This substrate is rarely degraded by a single force. In the survivor population, repetitive brain injury, post-traumatic stress, and depression each independently compromise the frontal pathways on which goal-directed behavior depends [28]. The strands remain conceptually distinct in that brain injury is not post-traumatic stress and post-traumatic stress is not depression, but they converge on the same neural real estate, where Saadi and colleagues have separately demonstrated independent contributions of brain injury severity and childhood trauma to neurobehavioral symptom burden [29]. The framework does not replace psychological understanding of intimate partner violence, nor does it treat psychological and neurobiological processes as competing alternatives; contemporary trauma science treats them as interdependent. It supplies the neurocognitive substrate that earlier frameworks left underspecified and accommodates the convergent etiology the survivor population actually presents, in which injuries, post-traumatic stress, and environmental constraints operate together. Where a survivor presents therefore matters as much as what she presents with, and Table 1 traces how the framework available in a given clinical setting shapes the attribution her symptoms receive.

These are characteristic defaults, not deterministic outcomes. The categories are heuristic rather than exclusive; trauma-informed clinical assessment routinely integrates psychological and neurobiological formulations, and the table is intended to show where a single dominant framework can bias attribution, not to suggest that clinicians working within psychological frameworks systematically miss injury.

### 1.4. Approach

This article is a narrative, conceptual review rather than a systematic review, and it makes no claim to exhaustive coverage. The literature was identified through iterative searches of PubMed, PsycINFO, Scopus, and Google Scholar across four thematic clusters: intimate partner violence and traumatic brain injury; mild traumatic brain injury mechanisms and hypoxic–ischemic injury; large-scale network disruption and traumatic brain injury; and the historical antecedents of concussive and trauma-related injury. Sources were selected for relevance and representativeness, with priority given to recent empirical work from 2023 to 2025, foundational studies, and the landmark or representative reports used to characterize each historical condition in the comparative tables. Because the synthesis is illustrative rather than comprehensive, no formal study-selection protocol or quality-assessment instrument was applied; this is acknowledged among the limitations below.

## 2. The Interpretive Vacuum

### 2.1. Brain Injury Behavior Is Poorly Recognized

The everyday behavioral manifestations of frontal-subcortical and network-level disruption remain widely misunderstood by clinicians and advocates, despite decades of research on traumatic brain injury. The neurocognitive functions most vulnerable to intimate partner violence-related trauma, including planning, cognitive sequencing, prospection, working memory, and valuation, are those that lack an intuitive observable signature [30,31]. Unlike focal motor deficits or language impairments, executive dysfunction is expressed through patterns of breakdown that are often interpreted through a psychological rather than neurological frame: disorganization, inertia, poor follow-through, or apparent inconsistency. Persistence of these symptoms is the rule rather than the exception: meta-analytic estimates place persistent post-concussion symptoms after mild traumatic brain injury at roughly one in six to one in three of general adult patients at three to six months post-injury [32], a baseline against which the survivor population, exposed repetitively and rarely surveilled, can only be assumed to fare worse.

Even within healthcare systems, these patterns are frequently misattributed to personality traits, emotional dysregulation, or ambivalence [6,19]. The result is a clinical environment in which the cognitive consequences of brain injury are often read as features of the person rather than features of the injury.

### 2.2. Psychological Explanations Fill the Vacuum

The psychological frameworks that have occupied this interpretive space were not wrong to identify what they observed. Walker’s learned helplessness framework [33,34], Dutton and Painter’s traumatic bonding theory [35], Beattie’s codependency framework [36], and related accounts of identification with the aggressor each described real phenomena in survivors. Each emerged in the absence of a competing biological account. Psychology stepped into an explanatory vacuum, providing internally coherent accounts of return to violent relationships and behavioral arrest that fit the data then available. This is what a responsive clinical science does when the injury mechanism has not yet entered clinical recognition.

What those frameworks could not do, given the tools available when they were developed, was distinguish between escape behavior that is fear-driven and escape behavior that is mechanically impaired by injury to the cognitive systems escape requires. The distinction matters because the interventions diverge sharply. A survivor whose difficulty leaving reflects psychological ambivalence responds to motivational and relational interventions. A survivor whose difficulty reflects impaired prospection, valuation, working memory, and multistep sequencing requires cognitive rehabilitation, accommodation of executive deficits in safety planning, and structured external scaffolding for the steps the brain can no longer hold together.

There is a point at which the psychological explanation does not merely become incomplete. When carotid compression has interrupted cerebral oxygenation or repeated rotational acceleration has produced diffuse axonal injury, an explanation that locates the difficulty in motivation or attachment style ceases to be a partial account and becomes a misattribution. Where neuroimaging documents these consequences directly, neurobiology is not an alternative reading. It is the reading that the prior frameworks were unable to access.

### 2.3. Secondary Harm from Misinterpretation

When survivors with intimate partner violence-related brain injury encounter psychological explanations for behaviors actually driven by injury-mediated impairment, the result is a secondary injury: a harmful reinterpretation of neurocognitive deficits as personal shortcomings. Survivors begin to question their own competence and character, interpreting impaired planning or decision execution as evidence that something is wrong with them rather than with the neural systems injured by the perpetrator’s violence [12,31].

The cost extends beyond the survivor. Without neurotrauma literacy, clinicians may read immobilization as motivational ambivalence or relational dependence rather than injury-driven incapacity [6,19]. Care plans are then built around the wrong target, and the survivor who would benefit from cognitive rehabilitation, accommodation, and external scaffolding receives instead interventions calibrated to a problem she does not have.

## 3. The Neuropsychological Entrapment Framework

This framework assigns causality exclusively to the perpetrator’s inflicted violence and provides a mechanistic account of behavioral constraints that locate impairment in the injury rather than the survivor. It does not pathologize survivors. It identifies the neurocognitive systems compromised by the inflicted injury and traces, step by step, how those compromises produce the apparent behavioral arrest that prior frameworks described in psychological terms.

### 3.1. Core Principle: Injury Constrains Agency

Difficulty leaving an abusive partner is often framed in psychological or relational terms. Yet survivors with intimate partner violence-related brain injury commonly demonstrate measurable impairment in the cognitive operations that escape requires: planning across multiple steps, holding alternatives in working memory while comparing them, simulating future states, valuing competing outcomes, and revising plans as circumstances shift. These cognitive operations are prerequisites for any sustained, goal-directed behavior in a hostile environment.

### 3.2. Effects of Intimate Partner Violence-Related Brain Injury on Cognitive Systems Necessary for Escape

The cognitive systems most vulnerable to intimate partner violence-related brain injury are the systems a survivor most needs to leave. Working memory loads required to coordinate finances, housing, transportation, and child safety in parallel exceed the capacity of survivors with measurable injury. Prospective memory failures interrupt the sequencing of planned departure.

Compounding this, the ventromedial prefrontal and hippocampal systems implicated in simulating future states are among those vulnerable to this injury pattern; where they are affected, the capacity to imagine where one would live, how the children would be enrolled in school, and what a safer life would concretely require may be correspondingly reduced. Without this simulation capacity, the present, however dangerous, can retain a structural coherence that the imagined future does not.

Frontal system disruption compounds these difficulties. Reduced cognitive flexibility, the capacity to revise plans as circumstances change, has been reported in women with intimate partner violence-related mild traumatic brain injury, though in samples that remain small and largely cross-sectional [10,11]. When the perpetrator’s pattern of violence shifts, when an opportunity for escape opens or closes, and when an external resource becomes available or vanishes, the cognitive operations required to update the plan may themselves be degraded.

What is often read as failure to act may, in this population, reflect injury to the cognitive processes that adaptive action requires rather than an absence of motivation. On this account, some of what presents as staying is better understood as the operational consequence of a degraded capacity to plan, sequence, and execute the multistep work that leaving demands. Agency is not absent; where injury is present, it may be constrained, alongside the substantial sociostructural, economic, and safety constraints that independently shape leaving. This is consistent with what shelter clinicians and advocates have long observed: Leaving is not a single decision but a sustained cognitive and material project. Repeated injury accumulates neurological burden [37], and the trajectory of violence often escalates, with rising severity calibrating lethality risk upward [38].

The pattern aligns with the competence-press framework Lawton and Nahemow formalized in gerontology [39], where adaptive functioning depends on the match between an individual’s cognitive capacities and the demands of the environment. Cognitive capacities have been reduced by injury at the moment the environment demands the most of them. Planning a way out, watching for the next escalation, and changing course when the situation changes are exactly the operations the injury has compromised. Adaptive functioning is constrained on both sides of the Lawton–Nahemow equation by reduced capacity and elevated demands. The clinical picture has not yet been formally characterized in this population, but what looks like an inability to adapt is consistent with the functional consequences of frontal-system impairment from this injury type.

### 3.3. Post-Traumatic Stress as a Neurobiological Force Multiplier

Post-traumatic stress does not explain staying through emotional attachment. It explains why injury-driven deficits become even more disabling. Hyperarousal, intrusive imagery, and threat-focused attention narrow the cognitive bandwidth available for planning. Sleep disruption, nearly universal in post-traumatic stress, degrades cognitive resilience and compounds processing speed and working memory deficits daily [40]. Saadi and colleagues provided direct empirical evidence that childhood trauma and intimate partner violence-related brain injury make independent additive contributions to neurobehavioral symptom burden in a shelter and community sample of survivors [29].

### 3.4. Summary

The neuropsychological entrapment framework reframes escape difficulty not as a manifestation of emotional dependency but as a structural and functional consequence of brain injury inflicted by the perpetrator. The framework locates responsibility in the source of the injury and reorients clinical and rehabilitative responses around the cognitive systems that need support.

## 4. Historical Pattern of Misattribution

Intimate partner violence-related brain injury is not the first condition in which the biological substrate of constrained behavioral capacity was misattributed to psychological causes, whether characterized as constitutional weakness in earlier eras or as somatization, functional disorder, or depressive equivalents in more recent ones. Across more than 150 years, medicine has repeatedly encountered injuries that the prevailing frameworks could not yet interpret and reached first for explanations that located the problem in the patient’s character rather than in the injuring mechanism. The conditions compared here differ materially in pathophysiology, evidentiary standard, and sociocultural and institutional context, and the parallels are offered as a heuristic rather than as evidence of an identical causal mechanism. Earlier frameworks are best understood not as simple failures but as rational responses to the diagnostic categories and tools then available. This section traces the recurring pattern with those caveats in view.

### 4.1. The Pattern in Prior Eras

The American Civil War produced over 600,000 military casualties yet left only modest traces of brain injury recognition in the medical record. Jacob Da Costa’s 1871 paper “On Irritable Heart” described persistent cardiovascular and somatic symptoms in soldiers, including palpitations, breathlessness, chest pain, and fatigue, that bore no consistent relationship to identifiable cardiac pathology [41]. The condition was attributed variously to constitutional weakness, irritable temperament, or excessive exertion. Da Costa’s syndrome was not strictly a head injury. It represented the symptomatic strand of combat trauma that emerged into the medical record above a much larger and largely unrecorded population of brain injuries sustained from gunshot wounds, artillery shrapnel, and blunt-force impact in the same casualties. The eventual recognition of these symptoms through the post-traumatic stress disorder construct established a neural substrate now understood to overlap mechanistically with that of combat-related mild traumatic brain injury [42]. Modern historiography traces the diagnostic lineage from irritable heart through soldier’s heart, neurocirculatory asthenia in World War I, and anxiety neurosis in World War II to the formal recognition of post-traumatic stress disorder in DSM-III [43,44,45]. McCrory and Berkovic document parallel conceptual drifts across the recorded history of concussion observation, with nineteenth-century clinicians struggling to localize the pathology of transient cognitive disturbance against prevailing models of cerebral function [46].

The pattern begins in the industrial era. Following the expansion of Britain’s railway network in the 1860s, physicians encountered patients who had survived train collisions but presented afterward with headaches, memory deficits, sensory disturbances, and persistent cognitive difficulty in the absence of visible injury. John Eric Erichsen attributed these complaints to microscopic molecular derangements of the spinal cord [47]. His mechanistic framing was challenged by Herbert Page, surgeon to the London and North West Railway, who proposed that fear alone, not physical injury, was sufficient to produce all described symptoms [48]. Page’s framing prevailed, and within decades the same survivor populations were being characterized as morally weak and suggestible [19]. The biological account did not return until the late twentieth century.

The Great War compressed this cycle. Charles Myers first described shell shock in 1915, initially noting its resemblance to hysteria before the sheer scale of presentations forced clinical reckoning with a population of soldiers whose exposure to high-explosive blast had produced cognitive, motor, and emotional disturbance [49]. Military medicine split by rank: Officers were managed in pastoral hospitals, while enlisted soldiers presenting with similar symptoms were court-martialed for cowardice and, in some cases, executed. The rank-stratified clinical response preserved existing social hierarchies while serving neither group’s recovery [19]. Lt. Col. F. W. Mott’s 1919 postmortem examinations of soldiers killed by blast revealed pulmonary hemorrhage, brainstem petechiae, and structural neural damage incompatible with the prevailing ‘functional disorder’ interpretation but did not settle the debate [50].

The same cycle repeated in athletics. Harrison Martland’s 1928 paper documented progressive cognitive and motor decline in boxers and proposed a causal role for repeated head trauma [51]. The term punch drunk was derisive enough that a gym owner later sued for defamation over its use, and the stigmatizing label attached itself to the clinical phenomenon for the next two decades [20]. Critchley’s reformulation introduced chronic traumatic encephalopathy as a clinical term in 1949, but rigorous pathological characterization arrived only with the late twentieth-century neuropathology literature, more than three decades after Corsellis and colleagues’ autopsy series identified tau protein deposition [20,52].

These histories illustrate what happens to available evidence when it implicates institutions with structural incentives to locate the origin of injury in the injured rather than in the injuring system. Casper’s analysis of the intertwined history of malingering and brain injury makes this case directly: Diagnostic recognition has tracked institutional interest, not biological reality [19]. This paper interprets that pattern through what it terms an institutional dynamic: Across diagnostic, administrative, and surveillance systems, recognition of harm has tended to organize around populations whose injuries existing frameworks were prepared to receive. We advance this as an interpretive reading of the historical record rather than as an established sociological finding. The history establishes a pattern of delayed recognition; it does not by itself establish that the same mechanisms produce the contemporary barriers examined below, which are documented on their own evidence.

So framed, the dynamic runs against the biological evidence: Where biology indicates inflicted injury, institutional structures have been slow to formalize it. The pattern is visible largely in retrospect, as interpretive frameworks have moved in step with the constituencies organized around affected populations. Parallel cycles can be traced in the long history of medicine’s psychogenic attributions for conditions later established as biological, until empirical discovery forced reframing. Table 2 assembles these cases, setting each condition’s initial attribution against its current understanding and the imaging technology available at the time.

Intimate partner violence-related brain injury is the only condition in this sequence where full diagnostic technology was available throughout the recognition period yet was not consistently applied. In every prior case, technological limitations provided at least a partial justification for delayed recognition. No such justification applies here.

### 4.2. IPV-Related Brain Injury Extends the Pattern

Intimate partner violence-related brain injury extends this pattern while also departing from it in one critical respect. It is the only condition in the sequence for which the full diagnostic neuroimaging suite, including computed tomography, magnetic resonance imaging, diffusion tensor imaging, and structural and functional connectivity analyses, has been continuously available throughout the period of non-recognition. Valera and Berenbaum published the foundational study in this literature, documenting that nearly three-quarters of battered women had sustained at least one partner-related brain injury, with severity negatively associated with memory, learning, and cognitive flexibility [11]. Neuroimaging confirmation of white matter disruption followed in 2018 [10]. The first dedicated autopsy series examining neuropathology in intimate partner violence-affected brains was not published until 2023 [53].

Psychological frameworks instead mapped the behavioral consequences of intimate partner violence-related brain injury onto culturally familiar categories of feminine weakness, dependency, and emotional dysregulation. Casper identifies the same mechanism across prior cases: When injury implicates a powerful institution, recognition is deferred not by absence of evidence but by absence of an institutional constituency willing to demand its inclusion in clinical frameworks [19]. Formal nosological absence should be distinguished from clinical awareness; interdisciplinary recognition of intimate partner violence-related neurotrauma has grown markedly across neuropsychology, rehabilitation medicine, neurology, and emergency medicine, even as the diagnostic frameworks have lagged.

## 5. The Contemporary Architecture of Non-Recognition

The historical pattern documented above persists in current clinical and administrative practice. Three levels are documented here: diagnostic criteria, administrative coding, and population surveillance. Each level reproduces the recognition gap through structural mechanisms that operate independently of clinical intent.

### 5.1. Diagnostic Criteria and the Person–Situation Problem

Whether a clinical presentation is recognized as brain injury or interpreted as emotional disturbance depends on more than the patient. From Lewin through Mischel, Endler and Magnusson, Bandura, and Mischel and Shoda, a half century of person-situation theory has established that behavior and the interpretive judgments rendered upon it are jointly produced by the actor and context [54,55,56,57,58]. For intimate partner violence-related brain injury, the relevant situation is the professional framework and organizational structure that receives the presentation and decides what it means.

The 2023 American Congress of Rehabilitation Medicine diagnostic criteria for mild traumatic brain injury, the field’s current nosological standard, do not enumerate strangulation-induced hypoxic–ischemic injury among qualifying mechanisms. The deliberative process that produced the 2023 revision was rigorous and international in scope [22,23], and the omission has a defensible scientific basis: The criteria were built around mechanical force transmitted to the brain, and strangulation injures through hypoxia rather than impact, a mechanism the traumatic brain injury construct was not designed to encompass. Hypoxic–ischemic injury has its own nosological standing, and nothing in the present argument requires that it be folded into a construct scoped to traumatic mechanisms. The omission is not an oversight, and the boundary it draws is coherent on its own terms.

The unresolved problem for this population lies elsewhere. Intimate partner violence commonly produces both mechanisms in the same survivor, and no current framework recognizes co-occurrence as a compound presentation rather than as two unrelated injuries. The broader landscape of competing frameworks (ACRM, VA/DoD, DSM-5-TR, ICD-11, and CISG) creates parallel diagnostic uncertainty that operates across mechanisms, with intimate partner violence-related brain injury and non-fatal strangulation among the cases that strain current criteria most directly [59]. Psychological trauma in these contexts co-occurs with brain injury rather than serving as an alternative explanation for it [22].

What remains situational is therefore not whether strangulation belongs within the traumatic brain injury construct but whether any professional constituency takes up the compound presentation as its concern. Criteria are extended when an organized constituency presses for a population’s inclusion, as military medicine and the Veterans Health Administration did for blast injury. No comparable constituency has yet formed around survivors who sustain impact and hypoxic–ischemic injury together.

Blast and strangulation are often discussed together, but the two mechanisms are pathophysiologically distinct. Blast injury is a mechanical, impact-type force; its effect on neural tissue resembles that of other traumatic brain injuries, acting on axons through pressure-driven shearing and stretch. Strangulation, by contrast, produces hypoxic–ischemic injury, depriving brain tissue of oxygen rather than imposing a mechanical load [7]; the traumatic brain injury construct was not designed to encompass this mechanism. Their shared feature is not pathophysiology but recognition history: Each lay outside the standard impact-based concussion framework, and each required population-specific advocacy to be considered at all. Blast injury entered the diagnostic criteria as a qualifying mechanism after the Operation Enduring Freedom and Operation Iraqi Freedom cohort produced sustained institutional pressure from military medicine and the Veterans Health Administration [42]. Blast qualified because it transmits mechanical force; strangulation does not, and its absence from the criteria follows from that difference. What institutional pressure determines is not where the traumatic brain injury boundary falls but whether the survivor who sustains injury on both sides of that boundary is recognized at all.

### 5.2. Administrative Coding Architecture

Administrative coding presents the next layer. The International Classification of Diseases, Tenth Revision, Clinical Modification provides traumatic brain injury codes within the S06 family for impact mechanisms and asphyxiation codes within the T71 family for hypoxic mechanisms, but it provides no means of registering their co-occurrence in a single patient. Each mechanism has its own code; what the architecture lacks is any way to record that a survivor of intimate partner violence sustained both, so the two injuries are documented as separate events rather than as the compound, dual-mechanism presentation they in fact represent. A clinician documenting a survivor with both head impact and strangulation must select between two coding pathways that capture only part of the clinical picture or distribute the documentation across two codes that the surveillance and reimbursement infrastructure will treat as separate problems.

The empirical consequences of this coding architecture have been documented directly. Tabaie and colleagues, examining 1,064,735 emergency department encounters at a Level I trauma center, found that International Classification of Diseases coding alone identified intimate partner violence cases at rates well below those obtainable from natural language processing of clinical notes [60]. Under the existing coding architecture, this population is hard to count. An undercounted population produces little surveillance signal, and without that signal, nosological revision rarely follows.

### 5.3. Population Surveillance Systems

Population surveillance systems carry the same structural limitation across multiple data architectures. The Centers for Disease Control and Prevention’s traumatic brain injury surveillance methodology categorizes external causes around firearms, motor vehicles, and falls [61]. Intimate partner violence is not a distinct surveillance category at the level the system was designed to capture.

Emergency medical services data carry parallel limitations at the point of first contact. AbiNader and colleagues document how emergency medical services clinicians enter International Classification of Diseases codes into National Emergency Medical Services Information System data fields, which means that the surveillance signal at the point of first contact is constrained by the same coding gap that operates at the hospital and registry level [26]. Appendix A sets out the relevant NEMSIS fields and the coding structure that produces this constraint.

Emergency department surveillance reveals the same pattern at the next clinical interface. Khurana and colleagues analyzed fifteen years of National Electronic Injury Surveillance System All Injury Programme data and found that intimate partner violence accounted for 40.4 percent of assault-related anoxia presentations and 31.9 percent of assault-related neck contusions [25]. Because the surveillance category structure does not separate intimate partner violence from other assault subtypes, these proportions, though striking, are not separable for downstream analysis and so do not translate into the population-level signal that drives nosological change. Table 3 extends the comparison across nosology, administrative coding, and population surveillance, reporting the lag interval for each condition.

These intervals measure the gap to mechanistically specific recognition by current standards. They should not be read to imply that the intervening period was empty: Earlier authorities frequently absorbed these presentations into broader categories, including encephalopathy and acute or chronic brain syndrome, and the lineage from punch drunk to dementia pugilistica to chronic traumatic encephalopathy illustrates how a general label was progressively specified. The interval therefore reflects evolution toward specificity rather than uniform non-recognition.

## 6. Neurobiological Substrates

The neuropsychological framework proposed in Section 3 rests on a specific neurobiological claim: that repetitive head trauma, strangulation-related hypoxic–ischemic injury, and chronic post-traumatic stress are hypothesized to converge on overlapping neural systems. These mechanisms differ materially in pathophysiology, chronicity, and likely clinical trajectory, and the evidence base for each in intimate partner violence populations remains preliminary; the convergence proposed here is a mechanistic hypothesis rather than an established unified pathology.

### 6.1. Repetitive Mild Traumatic Brain Injury

Repetitive mild traumatic brain injury produces diffuse axonal injury through rotational acceleration–deceleration forces that leave no reliable trace on conventional imaging but are detectable on diffusion tensor imaging. Valera and colleagues documented this pattern in women with intimate partner violence histories, finding a negative correlation between brain injury severity scores and fractional anisotropy in the posterior and superior corona radiata using diffusion tensor imaging [10]. Functional connectivity studies by the same research group have demonstrated reduced intrinsic functional connectivity between the right anterior insula and posterior cingulate cortex/precuneus, indicative of disrupted communication between salience and default mode networks central to attentional control and self-referential processing [27].

### 6.2. Strangulation and Hypoxic–Ischemic Injury

Nonfatal strangulation is mechanistically distinct from impact injury and is poorly captured by concussion frameworks designed around impact biomechanics. Compression of the carotid arteries produces transient cerebral hypoxia; venous obstruction produces intracranial pressure changes; combined mechanisms produce hypoxic–ischemic injury concentrated in watershed zones and metabolically vulnerable regions, including the hippocampus. Bichard and colleagues’ systematic review documented persistent neuropsychological sequelae of nonfatal strangulation [7]. Its long-term neurobehavioral consequences, including traumatic stress symptoms and self-reported vision problems persisting an average of nearly nine years after the most recent strangulation event, have only recently begun to receive systematic empirical attention [62].

### 6.3. Post-Traumatic Stress and Overlapping Neural Substrates

Post-traumatic stress in the context of intimate partner violence is not simply a psychiatric comorbidity. Amygdala hyperreactivity reduces the tonic prefrontal regulation required for executive operations. Hippocampal volume reductions, well documented in chronic post-traumatic stress, compound the hippocampal dysfunction produced by hypoxic injury. Sleep architecture disruption degrades the consolidation of new learning, including new safety planning. The result is that post-traumatic stress and brain injury operate on overlapping neural substrates and produce additive impairment in the cognitive systems escape requires [40].

### 6.4. The Convergence Point

What makes intimate partner violence-related brain injury mechanistically distinct from other recognized traumatic brain injury populations is not the presence of any single mechanism. It is the potential for simultaneous operation of repeated impact injury, strangulation-induced hypoxic–ischemic injury, and chronic post-traumatic stress in the same individual, often over years to decades, with each mechanism amplifying the others' effects on the same neural substrates.

The neuropathological picture that results is still being characterized. Dams-O’Connor and colleagues’ autopsy series, the first systematic examination of intimate partner violence-affected brains, found universal traumatic brain injury stigmata and microvascular pathology but no chronic traumatic encephalopathy meeting consensus criteria across either the initial or expanded series [53]. This absence should not be read as categorical exclusion. A small number of chronic traumatic encephalopathy cases have been documented in women who experienced intimate partner violence, and forensic-neuropathology commentary notes that consideration of the disorder is expanding to include this population [63]. The current literature suggests, however, that such cases represent the exception, arising with extreme chronic head trauma, rather than the norm. The present pattern nonetheless suggests that intimate partner violence-related brain injury is, in the main, neuropathologically distinct from CTE, characterized by combined impact, hypoxic, and stress-mediated injury rather than the tau-dominant pattern of repetitive subconcussive impacts.

### 6.5. From Neural Substrate to Behavioral Constraint

The preceding subsections document measurable disruption across cognitive systems for which intact function is a prerequisite for adaptive, multistep behavior in a hostile environment. The empirical literature now connects this neurobiological disruption to clinically observable cognitive impairment in survivors. Tanriverdi and colleagues found that intimate partner violence-related mild brain injuries were associated with lower visual memory scores in community-dwelling survivors assessed, on average, eight years after the violence ended [12].

Saadi and colleagues demonstrated that brain injury severity and childhood trauma contribute independently to neurobehavioral symptom burden, with injury severity specifically predicting cognitive subscale scores [29]. These findings mean that the cognitive entrapment framework rests on clinical realities already documented in the empirical literature. The framework names what the empirical findings imply for clinical practice and for the systems that deliver it.

## 7. Clinical Implications

The reframing proposed here has direct consequences for screening, neuropsychological assessment, rehabilitation planning, and the redesign of administrative and surveillance infrastructure. Recognition of intimate partner violence-related brain injury as a brain injury reorganizes clinical priorities.

Cognitive dysfunction in this population is rarely attributable to a single cause. Repetitive head trauma, non-fatal strangulation, post-traumatic stress, depression, sleep disruption, chronic stress, substance use, prior trauma, and socioeconomic adversity can each contribute, and disentangling their relative contributions empirically remains difficult. Cumulative head-impact exposure may also originate outside the intimate relationship, in childhood maltreatment, in sports, or from other trusted but non-intimate figures, and a complete history accounts for exposure within and beyond the index relationship. Although the literature centers on women survivors, intimate partner violence-related brain injury occurs across male and gender-diverse populations, and the cognitive account proposed here is meant to sit alongside, not above, the sociostructural, economic, and legal constraints that independently shape whether and how a survivor can leave. Survivors are not defined by the violence done to them; the framework addresses one injury-related constraint among the many dimensions of a full life.

Screening in domestic violence shelters, emergency departments, and primary care can draw on existing brain injury screening instruments, for example, the HELPS screen and the Ohio State University TBI Identification Method, while recognizing that the Boston Assessment of TBI-Lifetime has been adapted and validated specifically for this population as the BAT-L/IPV [64], although diagnostic thresholds remain to be established more broadly; mechanisms, including non-fatal strangulation, and cumulative exposure should be queried directly. Neuropsychological assessment in this population requires attention to the specific cognitive domains reported as vulnerable: executive function, prospection, working memory, and processing speed. The assessment battery should be sensitive enough to detect the patterns of subtle deficit that characterize repetitive mild injury, and the interpretation should account for the fragmentation of expertise across parallel service systems that rarely communicate. A survivor’s full clinical picture is rarely visible to any one clinician.

Rehabilitation planning, when grounded in the cognitive entrapment framework, addresses the cognitive systems escape requires rather than treating cognitive complaints as secondary to psychological symptoms. Cognitive rehabilitation approaches with demonstrated efficacy in other traumatic brain injury populations [65] are a reasonable starting point, but their effectiveness in intimate partner violence-related brain injury has not been established in controlled studies and is best framed as adaptation requiring evaluation rather than as settled practice. Any such adaptation requires attention to the specific ecology of the abuse environment, where rehabilitation gains must hold under conditions of ongoing threat, post-traumatic stress, and the structural barriers to safety planning that the survivor confronts.

The administrative coding and surveillance gaps documented in Section 5 are not merely bureaucratic problems. They are the mechanism by which the recognition gap is reproduced clinically and epidemiologically. Closing the gap requires changes at multiple levels: revision of diagnostic criteria to include the mechanisms characteristic of intimate partner violence-related brain injury; development of integrated coding for dual-mechanism injuries; and surveillance category structures that separate intimate partner violence from other assault subtypes. None of these changes is technically difficult. What each requires is institutional will.

## 8. Limitations and Research Agenda

Several limitations bound the claims advanced here. The cognitive entrapment framework is, at this stage, a conceptual proposal rather than a validated model; no study has yet demonstrated a direct, longitudinal link between specific neurocognitive deficits and real-world leaving behavior in this population. The neuroimaging and neuropsychological literature on intimate partner violence-related brain injury remains preliminary, characterized by small, heterogeneous samples and largely cross-sectional, correlational designs, and no imaging finding is yet a validated diagnostic biomarker applicable at the individual level. The neuropathological evidence rests on a single early autopsy program together with a small number of case reports. Cognitive impairment in survivors is multiply determined, and the contribution of brain injury cannot be cleanly isolated from post-traumatic stress, depression, sleep disruption, substance use, prior trauma, and socioeconomic adversity. Presentations are heterogeneous, and the analytic subtypes used here co-occur in practice. Consistent with a narrative review, no systematic quality assessment of the included literature was undertaken.

These limitations also define a risk in the opposite direction. Just as under-recognition has historically led to misattribution, premature or over-inclusive attribution of symptoms to brain injury could produce diagnostic overshadowing, minimize psychosocial contributors, or narrow therapeutic options. The framework is therefore intended to widen differential reasoning, not to supply a default explanation.

Framed as a set of predictions, the cognitive entrapment account becomes testable, and four directions follow directly. First, longitudinal designs can test whether injury-related executive and prospective-memory deficits, measured at intake, predict the trajectory and timing of leaving over follow-up, independent of post-traumatic stress and depression severity. Second, ecologically validated assessment, pairing performance-based measures of multistep planning and prospective memory with real-world functional indices, can test whether laboratory findings correspond to the specific safety-planning operations the framework implicates. Third, multimodal imaging in adequately powered samples can test whether the predicted salience and default-mode and frontal-systems disruptions track the proposed cognitive profile and distinguish intimate partner violence-related brain injury from post-traumatic stress alone and from other repetitive-impact populations. Fourth, dual-mechanism cohorts can test whether the combination of repetitive impact and non-fatal strangulation produces the additive impairment that the convergence hypothesis predicts, relative to either mechanism alone.

Each prediction is falsifiable, and each maps onto a limitation named above. The value of the framework, at this stage, lies less in what it establishes than in the specific, disconfirmable program of work it makes possible.

## 9. Conclusions

Medicine, when confronted with injury it cannot easily see, has a long record of misattributing the symptoms it can. This pattern repeats whenever an injury is invisible on conventional examination, whenever the mechanism is socially uncomfortable to acknowledge, and whenever the affected population lacks an organized constituency capable of forcing recognition into clinical language.

Intimate partner violence-related brain injury belongs in this sequence. It is the most recent instance of a pattern medicine has repeated and recognized after the fact for more than a century and a half. The distinction in this iteration is that the diagnostic methods required to characterize the injury were already established once the field turned its attention to it. The barrier has not been technological. It has been institutional and interpretive, located in the frameworks that determined whose injuries warranted clinical attention.

The neuropsychological entrapment framework proposed here is that competing framework. Rather than rejecting the psychological literature on intimate partner violence, it identifies the neurocognitive substrate that prior frameworks could describe behaviorally but could not mechanistically account for, and it locates responsibility in the perpetrator’s inflicted violence rather than in the survivor’s character or attachment style.

The sequence this paper has traced is straightforward. Recognition precedes diagnosis, diagnosis precedes treatment, and a population that clinical and surveillance systems do not reliably detect is one that those systems cannot consistently treat. The recognition gap and the treatment gap are not parallel phenomena; they are the same phenomenon, observed at different points in the clinical pipeline. There is little clinical justification for continuing to receive a brain-injured population principally through frameworks developed before the injury was recognized. The empirical foundation supports recognition, the technology has been sufficient for two decades, and what remains is the institutional decision to formalize what the data already indicate.

The institutional dynamic this paper has described as a mechanism of underservice is not static. In every prior iteration of this arc, recognition arrived only when an institutional constituency assembled around the affected population. Combat veterans of two world wars, professional athletes, and military personnel exposed to blast injury all generated the constituencies that ultimately produced their inclusion in clinical frameworks.

Constituencies for intimate partner violence-related brain injury are forming. The clinical literature is accumulating. The administrative and surveillance critiques are being articulated. The framework offered here is meant to contribute to the same project: to give the cognitive and behavioral consequences of intimate partner violence-related brain injury a clinically actionable name, grounded in the neuroscience of the injury rather than the psychology of the survivor.

## Figures and Tables

**Table 1:** How the receiving framework shapes default attribution.

Clinical Context	Available Framework	Default Attribution
Sports concussion clinic	mTBI/concussion literature	Symptoms → brain injury
Workers’ compensation/litigation	Secondary gain, malingering	Symptoms → motivation
Emergency department	Acute trauma protocols	Symptoms → injury (if visible)
Domestic violence shelter (1980s–2010s)	Walker, Beattie, trauma bonding	Symptoms → psychology
Domestic violence shelter (with IPV-BI framework)	Neuropsychological entrapment framework	Symptoms → brain injury

**Table 2:** Recognition lag and technology justification: comparing conditions.

Condition	Era	Initial Attribution	Current Understanding	Technology Available	Recognition Lag	Status
Soldier’s Heart/Da Costa’s	1862–1900s	Constitutional weakness, irritable temperament	Combat trauma, autonomic dysregulation; PTSD precursor	Limited clinical observation	~50 years (to shell shock)	Recognized (now PTSD)
Railway Spine	1860s–1890s	Hysteria, malingering	Spinal/CNS trauma	None	~30 years	Recognized
Shell Shock	WWI	Cowardice, moral weakness	Blast TBI + PTSD	None	~30 years	Recognized
Combat Fatigue	WWII	Psychological weakness	PTSD, TBI	None	~40 years	Recognized
Sports Concussion	1970s–1990s	‘Getting bell rung’	mTBI, axonal injury	CT, MRI (no DTI)	~25 years	Recognized
CTE	1928–2010s	‘Punch drunk’	Progressive tauopathy	Full suite (autopsy)	~77 years	Recognized
Blast TBI	1915–2003	Shell shock, combat fatigue, Gulf War syndrome	Diffuse axonal injury from blast overpressure	Mott 1919 (autopsy); CT 1971; MRI 1980s; full suite by 1990s	~88 years	Recognized (ACRM 2023 [22])
IPV-BI	1970s–present	Learned helplessness, codependency	TBI + HIBI + PTSD	FULL SUITE, not applied	40+ years	Ongoing

**Table 3:** Recognition lag across conditions: nosology, coding, and surveillance.

Condition/Era	Nosological Recognition	Administrative Coding	Population Surveillance	Lag Interval (Named → Mechanistic)
Soldier’s Heart/Da Costa’s Syndrome (1862–1900s)	Named: 1871 (Da Costa [41]). Mechanistic: 1980 DSM-III PTSD (Andreasen [43]).	PTSD codes from 1980.	PTSD captured in NCHS/SAMHSA from 2000s.	~109 years
Railway Spine (1860s–1890s)	Named: 1866 (Erichsen [47]). Mechanistic: misattributed 1885 (Page [48]); later subsumed under PTSD (Jones [45]).	Subsumed under PTSD or musculoskeletal codes.	Subsumed under PTSD surveillance.	~115 years
Shell Shock (WWI, 1914–1918)	Named: 1915–1918 (Myers [49]; Mott [50]). Mechanistic: subsumed under PTSD by DSM-III 1980 (Jones [45]; Andreasen [43]).	Subsumed under PTSD post-1980.	Subsumed under PTSD/military surveillance.	~62–65 years
Combat/Battle Fatigue (WWII, ~1943)	Named: ~1943 (Jones [45]). Mechanistic: DSM-III PTSD 1980 (Andreasen [43]).	Subsumed under PTSD post-1980.	Subsumed under PTSD/military surveillance.	~37 years
Sports Concussion (Late 20th c.–Present)	Named: late 20th c. (sport-concussion literature). Mechanistic: conceptual history [46]; ACRM mTBI updated 2023 [22].	mTBI within S06 family (ICD-10-CM).	NEISS sports injury surveillance from 1972.	~15 years
Chronic Traumatic Encephalopathy (1928–Present)	Named: 1928 (Martland [51]). Mechanistic: tauopathy characterization (McKee [52]); dementia pugilistica history (Castellani [20]).	ICD-11 partial entries (8E40 series).	Registry-based only (BU brain bank).	~93 years
Blast TBI (2003–Present)	Named: 2003 + OEF/OIF (Hoge [42]). Mechanistic: ACRM 2023 enumerates blast (Silverberg [22]).	S06 mTBI codes; no blast-specific code.	DoD/VA blast TBI registries (Hoge [42]).	~20 years
IPV-BI (1990s–Present)	Named: 1990s (limited IPV literature). Mechanistic: Not yet—no dedicated diagnostic framework (Iverson [37]).	Not yet—S06 + T71 separate; no IPV mechanism code (Tabaie [60]).	Not yet—IPV not separable in CDC, NEMSIS, NEISS (Coronado [61]; AbiNader [26]; Khurana [25]).	30+ years and counting

## Data Availability

No new data were created or analyzed in this study. All sources cited are publicly available through the journals and publishers identified in the reference list.

## Appendix A. NEMSIS Coding Architecture for Intimate Partner Violence-Related Brain Injury

The National Emergency Medical Services Information System (NEMSIS) was developed by the National Highway Traffic Safety Administration to provide a uniform national prehospital injury and care dataset [24]. The system houses more than 34 million events from over 10,000 emergency medical services agencies [26]. NEMSIS does not implement an injury coding system of its own. Emergency medical services clinicians enter International Classification of Diseases, Tenth Revision codes into specific data fields:

**Table A1:** NEMSIS data fields carrying ICD-10 codes.

Field	Description
eInjury.01	Cause of treated injury (ICD-10 coded)
eSituation.11	Primary clinical impression (ICD-10 coded)
eSituation.12	Secondary clinical impression (ICD-10 coded)
eDispatch.01	Dispatcher classification (categorical)
eScene.09	Scene location (categorical)

For an intimate partner violence-related brain injury patient with both traumatic brain injury and strangulation-induced hypoxic injury, the emergency medical services clinician must select two ICD-10 codes (S06 family for the impact injury, T71 family for the asphyxiation), neither of which captures the dual-mechanism nature of the injury. The selection occurs at the scene under time pressure and informs the dispatch classification, the primary clinical impression, and the secondary clinical impression that propagate through the receiving emergency department’s electronic health record.

The NEMSIS architecture inherits the dual-mechanism gap directly from ICD-10. There is no single code that designates the co-occurrence of both mechanisms in the context of intimate partner violence. The result is that the emergency medical services data system, which is the largest single source of pre-hospital injury surveillance in the United States, cannot generate the population-level signal required to force nosological revision of the diagnostic criteria that would, in turn, force the inclusion of the integrated mechanism in administrative coding. The architecture forms a closed loop that reproduces the recognition gap at every level.
